# Effects of bariatric surgery upon the sympathetic nervous system and hypothalamic-pituitary-adrenal axis in obese humans

**DOI:** 10.1038/s41598-025-14537-4

**Published:** 2025-08-07

**Authors:** Andreas Kraag Ziegler, Mette Christensen, Henrik Løvendahl Jørgensen, Mogens Fenger, Sara Fogh Myrup, Carsten Dirksen, Sten Madsbad

**Affiliations:** 1 Department of Clinical Biochemistry, Hvidovre University Hospital, University of Copenhagen, RegionH, Hvidovre, Denmark; 2 The Centre for Physical Activity Research, Rigshospitalet University Hospital, University of Copenhagen, RegionH, Copenhagen, Denmark; 3 Department of Endocrinology, Hvidovre University Hospital, University of Copenhagen, RegionH, Hvidovre, Denmark; 4 Department of Clinical Medicine, Faculty of Health and Medical Sciences, University of Copenhagen, Copenhagen, Denmark

**Keywords:** Gastric bypass, Bariatric surgery, Roux-en-Y Gastric Bypass, Weight loss, Autonomous nervous system, HPA-axis, Sympathetic nervous system, Metabolic health, Hormones, Cortisol, Metanephrines, Blood pressure, Endocrinology, Endocrine system and metabolic diseases, Metabolic syndrome, Obesity

## Abstract

Human obesity is a state of hyperactivity of the hypothalamic-pituitary-adrenal (HPA) axis and dysfunction of the sympathetic nervous system (SNS). It is unclear whether weight loss will normalize this apparent dysfunction and if potential changes are of short -or long-term duration. In this study, we test how weight reduction following Roux-en-Y Gastric Bypass (RYGB) surgery affects the HPA-axis and SNS activity for a follow-up period of 2 years. We show that a ≈ 30% reduction in bodyweight following RYGB, is accompanied by an increase in circulating cortisol, and decrease in the concentrations of systemic metanephrines concomitant with a transient reduction in systolic blood pressure, and that the endocrine changes persist for at least 24 months post-surgery. The decrease in SNS activity was weakly, but significantly correlated with postoperative improvements in HbA1c. These findings suggests that the anatomical rearrangement of the gastrointestinal system by bariatric surgery, and the resulting marked decrease in body weight, have long-term impact on the autonomic nervous system. The biological significance of these findings is uncertain though it could be speculated that chronically elevated serum cortisol, may be involved in the development of osteoporosis, a well-known risk of bariatric surgery, as well as control of glucose and lipid metabolism. Furthermore, the chronic reduction in metanephrines observed, suggest that SNS depression may contribute to both a reduction in blood pressure and better glycemic control following surgery. We collectively demonstrate that RYGB surgery has an early and persisting impact on the HPA-axis and the sympathetic nervous system, and that this is associated with bodyweight reduction and improved glycemic control.

## Introduction

Obesity is a constantly growing challenge with enormous economic consequences^1,2^. It is a multifaceted chronic disease with high comorbidity burden and increased mortality^3^. In conjunction with detrimental metabolic effects of obesity, the condition is also described as a state of endocrine dysregulation which may further worsen the metabolic dysfunction. Hence, obesity has been found to increase the activity of the sympathetic nervous system (SNS)^4,5^ and the hypothalamic-pituitary-adrenal axis (HPA-axis)^6–8^ which may contribute negatively to metabolic control. Accordingly, resolution of the obese condition through weight loss that leads to improved metabolism and overall health, may theoretically partly be related to normalization of the autonomic nervous system^9,10^. However, the existing literature on the regulation of the HPA-axis and SNS in response to lifestyle induced weight loss remain ambiguous^11^. Also, in many cases lifestyle intervention is insufficient to markedly reduce bodyweight, both short and long-term, owing in large part to non-compliance to the intervention^12^. Thus, for some individuals, bariatric surgery is the best choice for a permanent impact on metabolic health. Bariatric surgery, such as gastric bypass involves an anatomical rearrangement of the stomach and intestines. For the majority of patients, the procedure generally leads to a dramatic, fast and durable weight loss alongside improved metabolic control^13^. However, it remains scarcely explored^14^ how gastric bypass surgery and the accompanying weight loss will affect hormonal homeostasis of the SNS and HPA-axis during long-term follow-up, with existing literature showing ambiguous results.

The aim of this study is to explore whether weight loss induced through Roux-en-Y Gastric Bypass (RYGB) surgery impacts SNS and HPA axis activity, gauged by the amount of circulating catecholamine breakdown products (collectively denoted metanephines) and cortisol. In accordance, we describe changes in cortisol and metanephrines up to 2 years following surgery. We also explore whether changes in these stress and fight hormones are associated with HbA1c as a marker of glycemic control.

### Methods

This study included subjects from a prior single center cohort study from the Department of Endocrinology, Hvidovre -University Hospital, University of Copenhagen, Hvidovre, Denmark^15^. To summarize, patients suffering from severe obesity were subjected to Roux-en-Y Gastric Bypass (RYGB). Clinical and paraclinical data were then ascertained before surgery and up to 2 years following the surgical procedure. All participants had given written informed consent before participation, and the study was approved by the Scientific Ethics Committee of the Capital Region of Denmark (H-6-2014-029). The ethical approval conformed to the standards set by the Declaration of Helsinki and its later amendments. As we sought to explore prospectively the changes of hormones of the HPA-axis and SNS in detail, only subjects representing full datasets were included. Full data sets comprised serum samples from before surgery (baseline), and serum samples taken 3-, 6-, 12-, and 24-months (3 M, 6 M, 12 M and 24 M) following surgery. Clinical data including body weight and blood pressure were available from the same timepoints. Changes in Hb1AC were calculated with data from the original paper. In total 131 subjects fulfilled the criteria for full dataset acquisition and were subsequently included. Subject characteristics can be seen in Table 1.

**Table 1:** Baseline subject characteristics.

Parameter	Value
Age (years)	44.0 (± 9.5) (range: 22.4–64.0)
Height (cm)	171.6 (± 0.8) (range: 148–195)
Weight (kg)	126.0 (± 2.0) (range: 83.5-212.8)
BMI (kg/m^2^)	42.6 (± 9.2) (range: 32.5–64.9)
Systolic blood pressure (mmHg)	127.5 (± 1.2) (range: 100–175)
Diastolic blood pressure (mmHg)	81.1 (± 0.8) (range: 60–100)
Mean Arterial blood Pressure (mmHg)	96.6 (± 0.8) (range: 73.3-121.7)
Gender (% female)	67.1
Diabetes (%)	26.7

Data given as mean ± SD.

All samples were collected after an overnight fast between 7 and 10 AM. The serum samples were collected into EDTA vacutainers, centrifuged at 2500G at 4 °C for 10 min, allocated to cryotubes, and stored at −80 °C until further analysis. At the day of analysis, samples were gradually thawed in a lukewarm water bath over several hours.

#### Clinical data

Before inclusion, all participants underwent a thorough screening, encompassing socioeconomic factors, comorbidity, and medication status. Height was measured at baseline while bodyweight and blood pressure were measured at all visits. Diabetic status of the included subjects was ascertained by an endocrinologist at each visit according to IFCC HbA1c standard (HbA1c > 48 mmol/mol) or a history of diabetes with ongoing treatment with antidiabetic agents.

#### HPA-axis

Serum cortisol was measured utilizing the immuno-analysis through the Elecsys Cortisol II assay on COBAS 8000 (Roche Diagnostics).

#### Metanephrines

Serum samples (250 µL) were pre-treated with 225µL of 10 mM ammonium phosphate (buffered at pH = 6,5–6,7) and 25 µL of an internal standard working solution (40 nmol/L deuterium labelled metanephrine and normetanephrine). Pre-treated samples were loaded in individual wells of a Strata X-CW 96 Solid-Phase-Extraction (SPE) plate (Phenomenex, Torrance, USA) that had been preconditioned with 600 µL of 50:50 methanol/acetonitrile (MeOH/ACN) followed by 600 µL 10 mM ammonium phosphate (adjusted to pH = 6,5–6,7). After loading the samples, SPE wells were washed with 1500 µL of 10 mM ammonium phosphate (adjusted to pH = 6,5–6,7) followed by 1500-µL of 50:50 MeOH/ACN. Between washing steps, the SPE-well plate was dried under vacuum to remove as much solvent as possible from the sorbent bed. The target compounds were eluted from the plate with 300-µL 50:50 MeOH/ACN containing 5% formic acid into a 96-well sample collection plate, evaporated under N2 and resuspended in 250 µL mobile phase B. 10 µL of the eluate was injected onto a 3000 Ultimate liquid chromatograph coupled to an Altis TSQ mass spectrometer (ThermoFisher Scientific, Massachusetts, USA). An Acquity UPLC BEH Amide (1.8 μm; 2.1 × 100 mm) column, with an associated guard column, was used for chromatographic separation (Waters, Massachusetts, USA). Analysis was performed as previously described^16^ with slight modifications. In brief, elution occurred over 4 min with a gradient of 95:5 water: ACN containing 30 mM ammonium formate and adjusted to pH ≈ 3,0 (mobile phase A) and 15:85 water: ACN containing 30 mM ammonium formate with pH ≈ 3,0 (mobile phase B), a column temperature of 30 °C and a flow rate of 0.40–0.55 mL per minute. The gradient (expressed as %B) was as follows: 100% for 1 min at 0.4 ml/min; 100->90% linearly to 2.0 min at 0.4 ml/min; 90->70% linearly to 2.6 min at 0.55 ml/min; column recondition at 100%to 4 min at 0.55 ml/min. The autosampler was operated at 6 °C. The Altis TSQ mass spectrometer, used a Heated Electrospray Ionization (HESI) source. The following MS/MS transmissions (quantifier ions in bold) and parameters were utilized: Metanephrine 180->120/**148**; D3-Metanephrine 183->123/**151**; Normetanephrine 166->121/**134**; D3-Normethanephrine 169->124/**137**; Collision energy (V) = 18–19; Spay voltage; Positive Ion (V) = 2000; Vaporizer Temp = 350 °C; Ion Transfer Tube = 350 °C. More analytical details are available upon request.

#### Statistics

Statistical analyses were run in graphpad prism version 10.1 (GraphPad, San Diego). A 1-way ANOVA was applied to all datasets, and correlations were done using Pearson or Spearman correlation tests. Adjustment analysis for co-variates were done utilizing multiple linear regression. All data non-normally distributed, were log-transformed to apply parametric statistical testing. Statistical data are given as mean ± SEM unless otherwise stated, and significance was set to *p* < 0.05. HbA1c data from the original paper were included for correlative analysis only^14^.

## Results

### Subject preoperative characteristics

Preoperative subject characteristics are presented in Table 1. On average patients were middle-aged, predominantly women, and had class III obesity and a blood pressure within the normal range.

### Massive weight loss after RYGB surgery is accompanied by a long-term increase in circulating cortisol

**Fig. 1:**
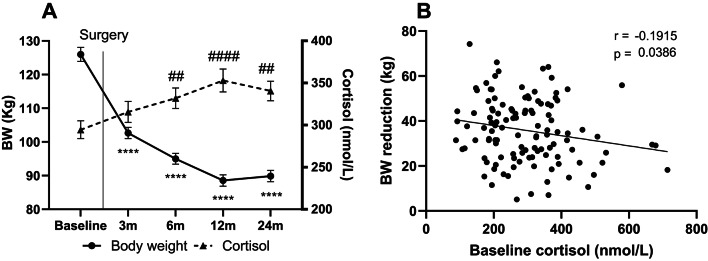
(**A**) Circulating serum cortisol (triangles) and body weight (circles) before (baseline) and 3–24 months after RYGB. (**B**) Correlation between baseline cortisol and the amount of bodyweight lost at 24 months following bariatric surgery. Data is given as mean ± SEM. ****p < 0.0001, ^#^*p* < 0.05, ^##^*p* < 0.01, ^####^*p* < 0.0001.

Before surgery the BMI was 42.6 ± 9.2 kg/m^2^. Gastric bypass surgery led to body weight reduction with a continuous decline until nadir at 12 months post-surgery (88.6 kg ≈ 30% reduction from baseline) (Fig. 1A). At 2-year follow-up, bodyweight was still ≈ 29% below baseline and mean BMI was 30 ± 0.5 kg/m^2^. Alongside the observed changes in bodyweight, circulating cortisol showed an inverse proportional pattern to body weight meaning that reduced bodyweight was associated with an increase in circulating cortisol of about 16% compared to before surgery (Fig. 1A). Curiously, even the small regain of bodyweight from 12 m to 24 m was linked to a corresponding decrease in cortisol, advocating for a very finetuned relationship between corporal mass and the effector hormone of the HPA-axis.

Furthermore, in correlation analysis, baseline cortisol was found to be a weak predictor of the degree of long-term weight loss following surgery (Fig. 1B). However, this association was no longer significant after adjusting for age, sex, and baseline bodyweight.

A sensitivity analysis including subjects with diabetes (*n* = 35) or subjects without antihypertensive medication (*n* = 67) showed the same trajectory for cortisol compared to the entire cohort, and that cortisol was not significantly associated to HbA1C neither in subgroup analysis nor for the entire dataset (SUPPL. Figure 1 A-C).

### Following RYGB surgery, levels of circulating metanephrines decreased, coinciding with a reduction in blood pressure and HbA1c

**Fig. 2:**
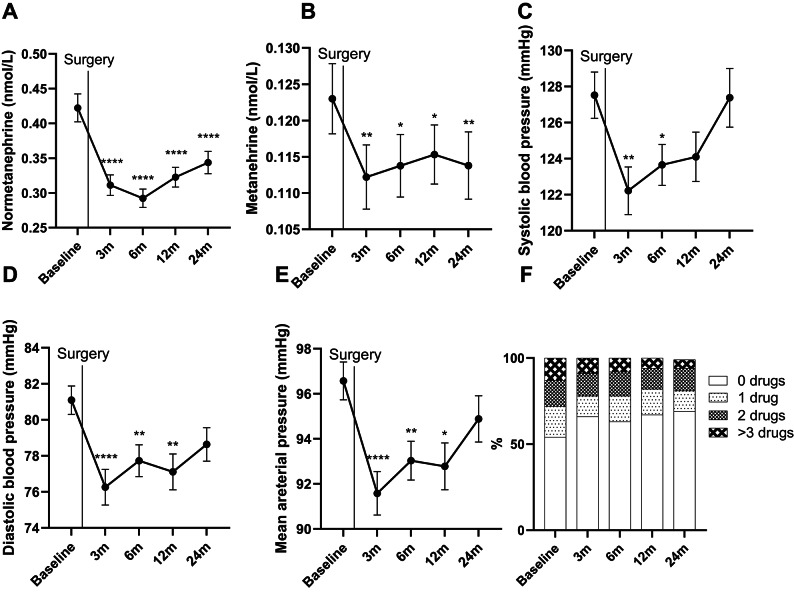
(**A**) Circulating serum normetanephrine. (**B**) Circulating serum metanephrine. (**C-E**) Systolic, diastolic, and mean arterial blood pressure. (**F**) Number of antihypertensive drugs used. Data given as mean and SEM. ****p < 0.0001, **p < 0.01, *p < 0.05.

**Fig. 3:**
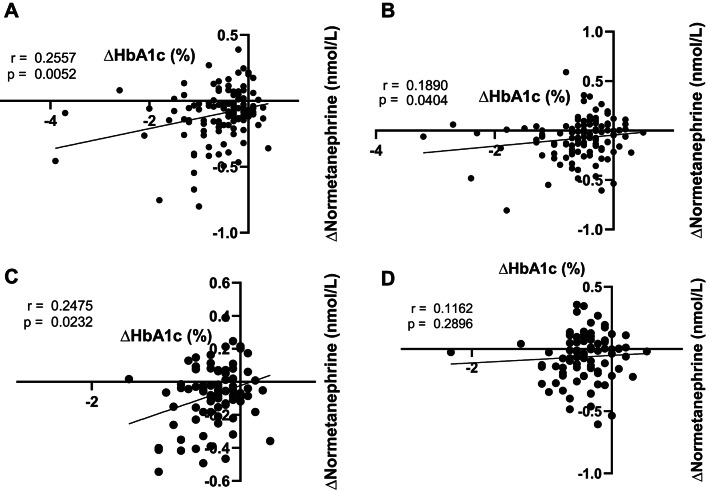
(**A**) Correlation between absolute changes in HbA1c (DCCT) and normetanephrine from baseline to 12 M (**A**) and 24 M (**B**) for the entire dataset (*n* = 131), and non-diabetics only (*n* = 96) (**C,D**).

RYGB led to a prompt and significant decrease in concentrations of metanephrine and normetanephrine, with values retained below baseline even at late follow-up at 12 and 24 months (Fig. 2AB). The observed changes in metanephrines coincided with a reduction in both systolic, diastolic, and mean arterial blood pressure (MAP) (Fig. 2.C-E). The lowest concentrations of metanephrines were observed at 3–6 months after surgery, with reduction in average concentration being more pronounced for normetanephrine (≈ 30% decrease from baseline) compared to metanephrine (≈ 10% decrease from baseline). The metanephrines and blood pressure changed in parallel with nadir of blood pressure after 3–6 months. Average systolic blood pressure increased after 3 months and did not differ from preoperative systolic blood pressure from 12 months and onwards, despite both normetanephrine and metanephrine still being significantly reduced compared with preoperative values. ≈46% of included subjects were taking 1 or more antihypertensive medications at inclusion, while follow-up period data showed that 2 years after surgery, only ≈ 30% were still on any antihypertensive medication (Fig. 2.F). Moreover, changes in normetanephrine and HbA1c at 12 and 24 months were significantly correlated both for the entire cohort (Fig. 3AB) and in patients without preoperative type 2 diabetes (Fig. 3CD).

As for cortisol, sensitivity analysis showed no distinct postoperative patterns in neither metanephrines nor blood pressure for subjects with or without diabetes nor subjects taking antihypertensive medications (data not shown).

## Discussion

Obesity is generally considered as state of autonomic endocrine dysfunction with increased drive on both the HPA-axis and the SNS, with proposed deleterious effects upon cardiometabolic health^4–6^. The current study sought to elucidate changes in HPA-axis and SNS activity following RYGB by assessing the prospective kinetics of cortisol and metanephrines, respectively, and if these hormones in turn could help predict long term weight loss and metabolic health. We found that the large weight loss, seen after surgery, coincided with an increase in the level of circulating cortisol (Fig. 1A), and a concomitant decrease in systemic normetanephrine, and to a lesser degree metanephrine (Fig. 2AB). This challenges the prevailing thought that correction of obesity will normalize activity of the HPA-axis and SNS. Furthermore, we found that the reduction in SNS activity correlated significantly to improvements in HbA1c, independent of diabetes medication, suggesting a possible mechanistic link between alterations in SNS activity following RYGB and improvements in glycemic control (Fig. 3A-D).

Aligning well with existing literature, we found that baseline cortisol levels were a weak predictor of long-term weight loss following RYGB^17^ (Fig. 1B). Accordingly, in theory, the higher the baseline cortisol, the less weight loss following bariatric surgery, can be expected. However, when adjusting for baseline bodyweight, sex and age, cortisol no longer predicted the expected weight loss after surgery, which was instead driven by male sex and baseline bodyweight. As a result, baseline cortisol is probably not a useful parameter for the estimation of predicted weight loss after bariatric surgery.

The rise in circulating cortisol we observe, upon visual inspection, shows an inverse relationship to weight lost, even 2 years following surgery, indicating a chronic increase in HPA-axis activity after RYGB (Fig. 1A). Hence, at 12- and 24-months follow-up, a relative increase in circulating cortisol of ≈ 20% and ≈ 16%, were found. Notably, after 24 months most of the participants were still overweight or obese with a mean BMI of 30 ± 0.5 kg/m^2^. The existing literature on cortisol and bariatric surgery is scarce and ambiguous, with some studies finding increased levels of cortisol while others find a reduction^18^. Hence, to date, no robust study has yet convincingly elucidated both short- and long-term kinetics of the endocrine profile in patients undergoing bariatric surgery. A study investigating blood glucose changes in response to a mixed meal test, found that even 4 years after RYGB surgery, meal induced hypoglycemia was not associated with an appropriate rise in counterregulatory blood cortisol in humans – indicating an autonomic and endocrine failure to maintain steady blood glucose^19^. The same study also noted a compensatory increase in nor-epinephrine from 60 min to 180 min following the meal ingestion. These findings underpin the notion that HPA axis and SNS regulation may be affected several years after bariatric surgery, supporting the data presented in the current paper.

To our knowledge, only 3 other studies show that bariatric surgery induces increased fasting cortisol concentration in the years after surgery. The first study was a small study that showed that in 24 women with obesity undergoing bariatric surgery, an increase in morning saliva cortisol was evident 6–12 months following the surgical procedure^20^. This study however, only collected 8 saliva samples at the 12-month timepoint and ended up combining data from 6- and 12-months making interpretation of the long-term changes in cortisol difficult. The second study suggested a decrease in Cortisol Binding Globulin (CBG) following biliopancreatic diversion with duodenal switch (BPD/DS), a surgical procedure characterized by more extensive malabsorption than RYGB and speculated that this would translate into increased levels of free cortisol in circulation 2 years after surgery^21^. However, total basal cortisol measured in that study did not show differences in circulating cortisol compared to age matched healthy controls. The third study found increased circulating cortisol following gastric bypass surgery, but only demonstrated this in a very small subset (11 out of 142 participants, all women) with the biggest weight reduction at 24 months following surgery, and did not statistically compare baseline, 12 months and 24 months^22^.

Thus, to date, aside from indications based on the mentioned studies, dynamic changes in serum cortisol following gastric bypass surgery remain largely unexplored. Hence, the current study represents the first robust human study in which changes in systemic cortisol, after bariatric surgery, are prospectively described.

The biological significance of an average increase in circulating cortisol of ≈ 16% at 2 year follow up is uncertain. Further, we are unable to say if this chronically elevated cortisol will decrease beyond the 2-year follow-up point. In support of long-term elevated cortisol following bariatric surgery, substantial bone loss 2 years after gastric bypass surgery have been reported^23^ and research have shown that even high endogenous cortisol within the normal range leads to increased skeletal attrition^24^. The increased risk of osteoporosis following bariatric surgery have traditionally been attributed to malabsorption of vitamin D and calcium as well as major weight loss^25,26^. However, despite having values within the normal ranges of vitamin D, calcium and parathyroid hormone (PTH), bone loss still occurs at an accelerated rate in patients subjected to bariatric surgery^23^. Although speculative, the current reporting of a chronically elevated cortisol levels 2 years following RYGB surgery may in fact contribute to the development of osteoporosis. It might also be speculated that the higher serum cortisol after major weight loss has impact on metabolic control, especially glucose metabolism and adipose tissue biology^27^ although the current study failed to demonstrate any link between circulating cortisol and HbA1c. Furthermore, it should be noted that Danish guidelines dictate that individuals seeking bariatric surgery must lose at least 8% bodyweight before being considered eligible for the procedure. Hence, the endocrine changes reported in the current paper should be interpreted in a context of the participants potentially already undergoing significant metabolic and endocrine adaptations.

Just as the long-term HPA-axis activity is yet scarcely described in relation to bariatric surgery, so is sympathetic nerve system activity. The available research would suggest, that circulating catecholamines decrease following weight loss, alongside blood pressure^4,28^. Further support of this notion can be found in a prior study investigating SNS activity in individuals with obesity before and after sleeve gastrectomy surgery, showing a decrease in muscle sympathetic nerve traffic (microneurography) 12 months post-surgery, while they did not directly measure systemic catecholamines^29^. These findings are congruent with the current study, showing a marked reduction in serum catecholamines for the duration of the follow-up period (Fig. 2AB). However, while existing literature would indeed predict a reduction in catecholamines following massive weight loss, it remains unclear how fast these changes occur, and how long they may last. Our study is uniquely suited to answer this question. Accordingly, we observe a robust reduction in serum metanephrines, predominately normetanephrine, as early as 3 months post-surgery, with levels below baseline, even at the 2-year follow-up point. This advocates for a chronic reduction in sympathetic nerve activity following RYGB surgery, which has not been documented in humans prior to this study. Lowered concentrations of metanephrines might partly explain the reduction in systolic and diastolic blood pressure observed until the 6 months follow-up for systolic and 12 months follow-up for diastolic blood pressure, likely owing to decreased vascular tone (Fig. 2C-E). The rebound of systolic blood pressure years after bariatric surgery has previously been demonstrated in the Swedish Obesity Study^30^. It should also be noted that antihypertensive drugs to a large extent are thought to reduce sympathetic nervous system activity and thus blood pressure^31^. However, in the current study, even despite a gradual reduction in hypertensive medication prescription (Fig. 2F), we observe a paradoxical decrease in circulating levels of metanephrines. It could therefore be speculated, that the theoretical reduction in sympathetic nervous system activity because of decreased antihypertensive drug use, is overruled by the sympathetic nervous system suppression caused by the massive weight loss itself.

Additionally, sympathetic nervous system hyperactivity has been postulated to contribute significantly to the metabolic syndrome, especially glycemic control^32^. Accordingly, data previously published from our dataset, have unsurprisingly, shown improved metabolic health, measured as glucose regulation, following the RYGB^15,33^. Therefore, it is very interesting to note that the reduction in SNS activity reported in this paper were in fact significantly correlated to HbA1c, the surrogate marker for metabolic/glycemic control. (Fig. 3). Thus, we propose a mechanism whereby chronic SNS suppression following RYGB surgery contributes to improved glucose regulation, while also acknowledging that this is almost certainly not the most prominent factor compared to the massive improvements in insulin sensitivity that often results from bariatric surgery.

The current study is of a descriptive nature limiting our ability to elucidate the mechanisms underlying the findings. Despite this, solid theoretical evidence would suggest that the decrease in catecholamines, could be explained by a reduction in circulating insulin, which is expected to occur following gastric bypass surgery^15^. Accordingly, insulin have been shown to stimulate sympathetic nerve activity in humans, resulting in increased amount of circulating norepinephrine^34,35^. Reduction in circulating insulin does not readily explain the data for the HPA-axis, however, as insulin has been shown to induce HPA activity and, de facto, cortisol release^34,36^. Another hormone that decreases following bariatric surgery, is leptin^37^. Intriguingly, leptin has been shown to inhibit HPA-axis activity^38,39^ while simultaneously stimulating the sympathetic nervous system output^40,41^Thus, a bariatric surgery-induced drop in circulating leptin, would indeed predict decreased circulating amounts of catecholamines, and a concomitant increase in systemic cortisol, which is exactly what is reported in the current study. This however remains a theoretical consideration since we are not aware of any study reporting the combined interrelation between reduction in circulating leptin and changes in the HPA-axis activity and sympathetic nervous system output.

In conclusion, the present study is a robust and well powered study that challenges the prevailing notion of obesity as an endocrine dysfunction, that upon correction, normalizes both HPA axis and SNS activity. The currently presented human data shows that RYGB surgery facilitates a large reduction in bodyweight and a moderate reduction in blood pressure and HbA1C. These expected findings are accompanied by a gradual and lasting increase in circulating cortisol and a simultaneous reduction in systemic metanephrines, suggesting a divergent impact on the HPA axis and sympathetic nerve activity. Further studies are needed to understand the mechanisms behind these changes, in particular the role of leptin is an interesting future avenue. From a clinical point of view, the reduced SNS activity observed may partly pertain to better glycemic control, and reduction in blood pressure, while the long-term elevation in cortisol may theoretically pose a risk for the development of osteoporosis.

### Strengths and limitations

The current study represents a relatively large single-center cohort with frequent postoperative follow-up for up to 2 years with matched clinical and biochemical data. Accordingly, this allows for a detailed description of the kinetics of the effector hormones of the HPA-axis and sympathetic nervous system matched to clinical data. We have included data on antihypertensive drug use since this class of drugs may impact the sympathetic nervous system while the effects upon the HPA-axis are largely unknown.

The study, being completely descriptive in nature, relies on correlation analysis and theoretical framework for mechanistic considerations, and thus cannot directly address cause and effect. Also, we acknowledge that the lack of a matched control group is a limitation, but inclusion of an obese control-group would have been an unethical approach since severely obese subjects are considered severely ill. Further, we acknowledge that the time of day impacts the level of both HPA-axis and SNS activity, and that strictly standardized sampling times would have been the preferred option. However, we have largely mitigated this variation by obtaining fasted blood samples in the noon between 7 and 10 pm, which, in our eyes is an acceptable standard within clinical research.

Finally, the current study did not investigate at what level the observed changes in HPA-axis activation were affected by RYGB surgery. Thus, we are not able to pinpoint if the altered HPA-axis activity is originating at the hypothalamus (Corticotropin Releasing Hormone/CRH) or pituitary gland (AdrenoCorticoTropic Hormone/ACTH).

## Supplementary Information

Supplementary Information 1.

## Data Availability

The datasets generated during and/or analyzed during the current study are not publicly available but are available from the corresponding author on reasonable request.

## Declarations

### Inclusion and diversity

We support inclusive, diverse, and equitable conduct of research.
