# Supplementary material for: Effects of bariatric surgery upon the sympathetic nervous system and hypothalamic-pituitary-adrenal axis in obese humans
Source: Sci Rep. 2025 Aug 7;15:28916. doi: 10.1038/s41598-025-14537-4 (PMC12332009; doi:10.1038/s41598-025-14537-4)
Supplement: Supplementary file 1 — Supplementary Information 1. [file 41598_2025_14537_MOESM1_ESM.docx]

**SUPPL. FIG.1:** A-B**)** Circulating cortisol in subjects without antihypertensive treatment (n=67) or with diabetes (n= 35) respectively. C) Correlation between absolute changes in HbA1c (DCCT) and cortisol from baseline to 12M for the entire dataset (n=131). Data given as mean and SEM. **=p<0.01, *=p<0.05. Inserts represents p-values.
